# Comparison of Immune Responses Elicited by Ad5F35-AEgp145 Alone or in Combination with rMVA-AEgp145

**DOI:** 10.3390/v18010079

**Published:** 2026-01-06

**Authors:** Jing Yang, Qi Ma, Xiaozhou He, Hongxia Li, Xiaoguang Zhang, Yanzhe Hao, Xia Feng

**Affiliations:** National Key Laboratory of Intelligent Tracking and Forecasting for Infectious Diseases, National Institute for Viral Disease Control and Prevention, Chinese Center for Disease Control and Prevention, Beijing 100052, China; yangjing@ivdc.chinacdc.cn (J.Y.); 13179818951@163.com (Q.M.); hexz@ivdc.chinacdc.cn (X.H.); lihx@ivdc.chinacdc.cn (H.L.); zhangxg@ivdc.chinacdc.cn (X.Z.)

**Keywords:** HIV vaccine, homologous and heterologous immunization strategies, replication-deficient viral vectors

## Abstract

Background: Developing an effective vaccine is crucial for the prevention and control of AIDS. Viral vector-based vaccines, particularly those utilizing homologous or heterologous prime-boost strategies, represent an important direction in current HIV vaccine research. Methods: In this study, replication-defective chimeric adenovirus Ad5F35 and modified vaccinia virus Ankara (rMVA) vector vaccines expressing the HIV-1 AEgp145 were successfully constructed, designated as Ad5F35-AEgp145 and rMVA-AEgp145, respectively. Sixty BALB/c mice were randomly divided into three groups: Ad5F35 alone, rMVA prime/Ad5F35 boost, and PBS control. The mice were immunized intramuscularly at weeks 0 and 3, and humoral and cellular immune responses were assessed at 4, 8, 12, and 16 weeks after the initial immunization. Results: The homologous Ad5F35 and heterologous rMVA/Ad5F35 vaccination regimens elicited comparable levels of HIV Env-specific cellular immune responses, peaking at 2100 ± 222 SFCs/million splenocytes and 2200 ± 619 SFCs/million splenocytes, respectively (*p* > 0.05). Compared to the heterologous regimen, the homologous Ad5F35 regimen induced significantly higher levels of gp120-binding antibodies at weeks 4 and 8 post-initial immunization, with geometric mean titers of 1:25,600 ± 7011 versus 1:1280 ± 150.7 and 1:10,240 ± 4048 versus 1:2560 ± 391.9, respectively. Furthermore, neutralizing activity at week 8 was significantly higher in the homologous group, with a 50% neutralization titers of 1:45 compared to 1:12 in the heterologous group (*p* < 0.01). Conclusion: This study demonstrates that the Ad5F35-AEgp145 vaccine, whether administered alone or in combination with rMVA-AEgp145, effectively induces strong and comparable cellular immune responses targeting HIV-1 Env in mice. While both regimens are effective, homologous immunization elicits moderately higher levels of antibody responses. These findings provide an important foundation for the further investigation of vector-based HIV vaccine formulations.

## 1. Introduction

HIV-1, the causative agent of AIDS, was identified in the early 1980s and has turned into a global epidemic [1]. While antiretroviral therapy (ART) has greatly decreased AIDS-related morbidity and mortality worldwide, substantially improving the life expectancy of people living with HIV [2], the expanded access to ART has inevitably led to the emergence of drug-resistant strains, compromising treatment efficacy [3]. Therefore, developing effective HIV vaccines remains crucial for prevention and control of HIV infection.

HIV is a retrovirus with a single-stranded RNA genome. The main challenges in HIV vaccine research include the following: 1. The virus exhibits an extremely high mutation rate, resulting in exceptional genetic diversity. This leads to the formation of numerous subtypes and circulating recombinant forms (CRFs), making HIV highly prone to immune escape [4]. 2. During its replication cycle, proviral DNA integrates into the host genome, establishing a latent infection reservoir that evades detection and clearance by the immune system [5]. 3. Since humans are the only natural hosts for HIV, the absence of appropriate animal models for challenge studies poses a major constraint on HIV vaccine development.

In addressing these challenges, viral vector vaccine technology has emerged as a key strategy in HIV vaccine development due to its ability to effectively elicit cellular immune responses. Viral vector-based vaccines have demonstrated promising results [6,7,8]. These vaccines are developed by integrating the target gene into the viral vector genome. Upon administration, they infect host cells and drive the expression of the target antigen, thereby inducing antigen-specific immune responses in vaccinated animals [9,10]. The immune responses elicited by homologous (same vector) or heterologous (different vectors) prime-boost immunization strategies exhibit distinct characteristics [11,12]. Among these vectors, replication-deficient adenovirus serotype 5 (Ad5)—which carries deletions in the essential replication genes E1 and E3—and replication-deficient modified vaccinia virus Ankara (MVA) have been shown to induce strong cellular immune responses and provide high level of protection in non-human primates [7,8]. Although Ad5 is a potent vector, its widespread seroprevalence may limit its clinical application [13]. To address this limitation, researchers have developed alternative adenovirus serotypes and chimeric adenovirus vectors. One such example is the Ad5F35 vector, a chimeric adenovirus created by replacing the Ad5 fiber protein with that of adenovirus serotype 35 (Ad35) [14]. This modified vector retains efficient gene delivery capability even in the presence of pre-existing Ad5-neutralizing antibodies [15]. Ad5F35, as a vaccine vector, can induce robust antigen-specific CD8^+^ and CD4^+^ T cell responses, thereby achieving broad immune activation. Because pre-existing neutralizing antibodies against Ad35 are rare in the human population, Ad5F35 can effectively evade immune clearance directed against the commonly used Ad5 vector, resulting in enhanced vaccine potency. Moreover, it exhibits more favorable in vivo tropism, with markedly reduced accumulation in the liver compared with Ad5, thereby lowering the risk of inflammation and hepatotoxicity and improving overall safety. These features make Ad5F35 an attractive platform for the development of antiviral and antitumor vaccines, as well as for constructing prime–boost immunization strategies, particularly in settings where strong cellular immune responses are required. Modified vaccinia virus Ankara (MVA) is a member of the genus *Orthopoxvirus* within the family *Poxviridae*. As a vaccine platform, it offers several unique advantages. First, it is a highly attenuated, non-replicating poxvirus that has lost the ability to replicate its genome in human and most other mammalian cell lines, cannot generate infectious viral particles, and can only be propagated in a limited number of specific permissive cell lines [16]. Second, its large genome provides ample capacity for the stable insertion and efficient expression of large exogenous antigen-encoding sequences. In HIV vaccine development, MVA vectors expressing different antigens have shown an excellent safety profile and strong immunogenicity in clinical trials [17,18,19]. Notably, MVA has no documented history of natural infection in humans, effectively precluding interference from pre-existing vector-specific immunity.

The HIV viral particle consists of an outer lipid envelope, a middle capsid protein layer, and an inner viral core. The HIV envelope is sparsely embedded with envelope glycoprotein trimers. The glycoprotein precursor gp160, encoded by the env gene, is cleaved by host proteases into surface protein gp120 and transmembrane protein gp41, which together form the envelope glycoprotein trimer. In this study, the immunogen AEgp145 was specifically designed as a key vaccine antigen targeting the predominant HIV strain circulating in China (CRF01_AE subtype). It is based on the complete envelope glycoprotein precursor gp160 of this subtype, with its C-terminal intracellular domain truncated, resulting in a protein of approximately 145 kDa, designated gp145. This truncation retains key immunogenic structures—including the major portions of gp120 and gp41—while significantly reducing the cytotoxicity associated with the full-length native protein [20]. Furthermore, its gene sequence has been codon-optimized to enhance expression efficiency in human cells. Therefore, AEgp145 represents a safety-modified and expression-optimized vaccine antigen designed to better mimic the viral surface and elicit protective neutralizing antibodies and T-cell immune responses. Our previous research has shown that the truncated Env antigen gp145 exhibits stronger immunogenicity compared to the full-length gp160. Specifically, prior studies have already employed CRF01_AE (CM244) gp145 as a vaccine immunogen and compared gp145-based regimens with alternative Env designs in nonhuman primates, including regimens explicitly using CM244 gp145 DNA and reporting recognition of membrane-bound trimeric gp145 Env from clade AE.CM244 [21]. In addition, related follow-up work further discusses and builds on these CM244 gp145 DNA–containing regimens in the context of directing antibody responses [22]. Beyond AE specifically, membrane-bound trimeric gp145 displayed on the surface of infected/transduced cells and delivered by viral-vector platforms has also been characterized and evaluated as a vaccination strategy, providing important background on “membrane-bound gp145” approaches [23].

The HIV envelope immunogen design has evolved significantly toward structurally stabilized trimers that preserve native-like antigenic conformation and glycosylation patterns. Beyond membrane-bound designs, soluble uncleaved gp145 constructs represent an important parallel approach within the contemporary landscape of HIV vaccine development. The uncleaved gp145 immunogen retains the trimeric architecture and incorporates key structural domains including the membrane-proximal external region (MPER), while offering improved manufacturability and safety profiles compared to full-length gp160. Recent comparative studies have demonstrated that gp145 produced in mammalian systems maintains native-like antigenicity, trimer stability, and appropriate glycosylation profiles, supporting its potential as a clinical-stage vaccine candidate [24,25].

In this study, we used two replication-deficient viral vectors: a chimeric adenovirus type 5 vector with type 35 fiber (Ad5F35) and recombinant modified vaccinia virus Ankara (rMVA). The two viral vector-based vaccines expressing HIV-1 AEgp145 were constructed, respectively, and their immunogenicity was evaluated in mice.

## 2. Materials and Methods

### 2.1. Experimental Materials and Cells

Mouse lymphocyte separation medium was obtained from Dakewe Biotech Co., Ltd. (Beijing, China). Mouse IFN-γ ELISpot kits were obtained from Mabtech (Stockholm, Sweden). Phorbol 12-myristate 13-acetate (PMA) and ionomycin were purchased from Sigma-Aldrich (St. Louis, MO, USA). HIV-1 subtype AE pseudovirus, BHK-21 cells, HEK293T cells and TZM-bl cells were stored in the laboratory of Academician Zeng Yi at the National Institute for Viral Disease Control and Prevention, Chinese Center for Disease Control and Prevention. The H-2d-restricted HIV-1 (AE) Env-specific peptides were synthesized by ChinaPeptides (QYAOBIO, Suzhou, China). The 2G12 antibody (a CHO-derived recombinant human monoclonal antibody against HIV-1 gp120) and HIV-1 AE consensus gp120 protein were purchased from Cambridge Biologics (Boston, MA, USA).

### 2.2. Construction of Ad5F35 and rMVA Vector HIV Vaccines

**Figure 1:**
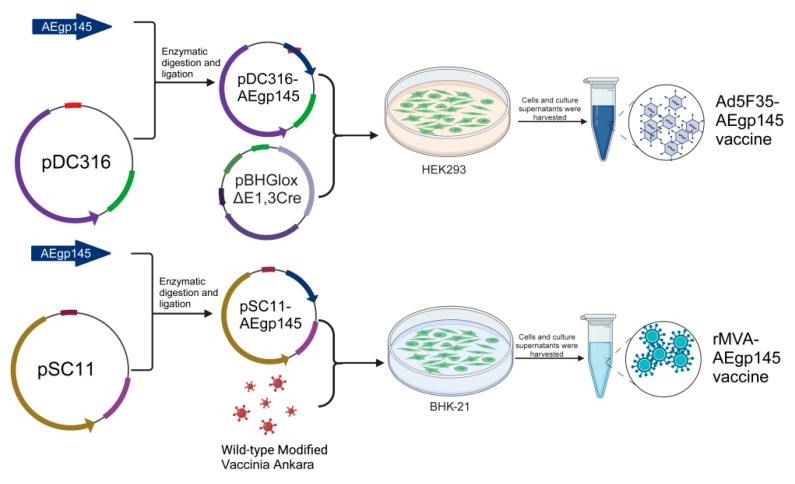
Schematic architecture of recombinant HIV vaccines: chimeric adenovirus type 5 vector with type 35 fiber (Ad5F35), recombinant modified vaccinia virus Ankara (rMVA) engineering strategies.

The env gene of HIV-1 CRF01_AE was codon-optimized in our laboratory, with the optimized sequence synthesized by Invitrogen Corporation (Shanghai Representative Office, Shanghai, China). PCR amplification was applied to introduce a termination codon immediately after the extracellular region of the AEgp41 protein (at amino acid position 695), generating the AEgp145 gene that lacks the intracellular region and part of the transmembrane domain. The modified AEgp145 gene was inserted into the pSC11 vector for recombinant virus construction. BHK-21 cells infected with wild-type MVA were transfected with pSC11-AEgp145, and the recombinant virus rMVA-AEgp145 was produced through homologous recombination. Amplification and purification of the recombinant MVA vaccine were conducted by Shenzhen Yuanxing Genetech Co., Ltd. (Shenzhen, China), yielding a final titer of 1.27 × 10^9^ PFU/mL. A recombinant adenovirus Ad5F35-AEgp145 carrying the identical AEgp145 gene was also generated. Its packaging, amplification and purification were completed by Beijing WuJiaHe Gene Technology Co., Ltd (Beijing, China)., with the resulting titer reaching 6.3 × 10^8^ PFU/mL (Figure 1). All vaccine preparations were aliquoted and stored at −80 °C for subsequent use.

### 2.3. Animals

Sixty female BALB/c mice, aged 4 to 6 weeks, were purchased from SPF (Beijing, China) Biotechnology Co., Ltd. and housed in the animal laboratory of the Institute of Occupational Health and Poison Control, Chinese Center for Disease Control and Prevention. The mice experiment was reviewed and approved by the Ethics Committee for Experimental Animals of the National Institute for Viral Disease Control and Prevention, Chinese Center for Disease Control and Prevention (approval number: 20200612027).

### 2.4. Identification of Exogenous Proteins Expressed in Recombinant Viruses

BHK-21 cells, HEK293T cells and TZM-bl cells were stored in the laboratory of Academi-cian Zeng Yi at the National Institute for Viral Disease Control and Prevention, Chinese Center for Disease Control and Prevention. HEK293 cells were seeded in 6-well plates at a density of 2 × 10^5^ cells per well, and BHK-21 cells were seeded at a density of 4 × 10^5^ cells per well. All cells were cultured in a humidified incubator at 37 °C with 5% CO_2_ for 24 h. Subsequently, HEK293 cells were infected with 1 × 10^6^ plaque-forming units (PFU) of Ad5F35-AEgp145, while BHK-21 cells were infected with rMVA-AEgp145 at a multiplicity of infection (MOI) of 0.1. Uninfected control wells were included. Cells were collected 24 h post-infection and the expression of HIV-1 Gp145 protein was analyzed by SDS-PAGE and Western blotting. Proteins were separated by SDS–PAGE on a 8% polyacrylamide gel under reducing conditions at 150 V for 50 min, and subsequently transferred to a PVDF membrane for immunoblotting. All blot images were visualized using the Tanon-4800Multi system (Shanghai, China).

### 2.5. Transmission Electron Microscopy (TEM) Analysis of Recombinant Viruses

The morphology of rMVA and Ad5F35 recombinant viruses was analyzed by transmission electron microscopy (TEM, Thermo Fisher Scientific, Waltham, MA, USA) using negative staining. Briefly, a 100 µL aliquot of the 1:10 diluted virus suspension was applied to the carbon-coated side of a copper grid. Excess sample was removed by wicking the grid’s edge with filter paper. The grid was then inverted onto a drop of 2% phosphotungstic acid (pH 6.5–7.0) for 0.5–2 min. After staining, the grid was lifted, and residual liquid was blotted with filter paper. Following air-drying (1–3 min), viral morphology was visualized by TEM. Mouse Immunization and Detection Protocols

### 2.6. Mouse Immunization and Immune Response Detection Protocols

**Figure 2:**
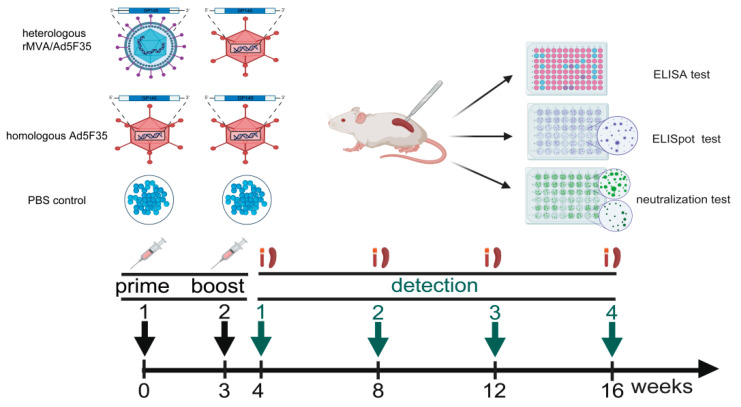
Immunization and sampling timeline. Mice in the homologous Ad5F35 HIV vaccine group received a prime immunization with Ad5F35-AEgp145 at week 0. Mice in the heterologous rMVA/Ad5F35 HIV vaccine group received a prime immunization with rMVA-AEgp145 at week 0. Both groups received an Ad5F35-AEgp145 booster immunization at week 3 (10^6^ PFU/dose/mouse). Sera and splenocytes were isolated at weeks 4, 8, 12, and 16 for analysis.

The 60 mice were randomly divided into three groups (*n* = 20 per group): homologous Ad5F35 vector HIV vaccine, heterologous rMVA/Ad5F35 vector HIV vaccine, and PBS control groups. Immunizations were administered via intramuscular injection into the anterior tibial muscle at weeks 0 and 3 (10^6^ PFU/100 μL per mouse). Serum samples were collected at 4, 8, 12 and 16 weeks post-primary immunization and stored at −20 °C for antibody detection. Splenic lymphocytes were isolated using a mouse lymphocyte separation solution for cellular immune response assays (Figure 2).

### 2.7. Enzyme-Linked Immunosorbent Assay (ELISA)

The test sera were heat-inactivated at 56 °C for 30 min and subsequently aliquoted for use. The levels of HIV-1 Gp120-binding antibodies in the sera of immunized mice were quantified using an indirect ELISA. Briefly, purified HIV-1 Clade AE gp120 antigens (Immune Technology, San Diego, CA, USA) were coated onto 96-well microtiter plates at a concentration of 100 ng/100 μL/well and incubated overnight at 4 °C. The wells were blocked with PBS containing 5% skim milk for 2 h at 37 °C. Serially diluted sera samples were added and incubated for 1 h at 37 °C. After washing the plates five times, 100 μL of 1:20,000 diluted HRP-conjugated goat anti-mouse IgG antibodies (Zhongshan Golden Bridge, Beijing, China) was then added. The plates were incubated at 37 °C for 1 h. Subsequently, the samples were developed with TMB (WanTai, Beijing, China) at 37 °C for 30 min. The reactions were stopped using 1 M H2SO4 and the absorbance was measured at 450 nm with a reference wavelength of 630 nm (BioRad, Hercules, CA, USA). Each plate included positive control sera, negative control sera, blank wells, and internal quality control samples. All controls and sera samples were assayed in duplicate. The endpoint titer was defined as the reciprocal of the highest serum dilution with an OD450 value exceeding the assay cut-off. The cut-off value was calculated as the mean OD450 + 0.15 (approximately 3 SD) of eight negative control sera at the 1:100 dilution. Samples negative at the starting dilution were assigned a titer of 1:50 for analysis. Assay precision was validated; intra-assay CVs (*n* = 10) and inter-assay CVs (*n* = 5) were <10% and <15%, respectively.

### 2.8. Pseudovirus Based Neutralization Assay

TZM-bl cells in optimal condition were seeded into 96-well culture plates at a density of 5 × 10^3^ cells per well. Two-fold serially diluted test sera, starting from 1:10 dilution, were prepared using complete DMEM culture medium. The subtype AE HIV-1 pseudovirus prepared in our laboratory was diluted to a concentration of 400 TCID50/ml with complete DMEM culture medium. Fifty microliters (50 µL) of diluted serum sample was mixed with an equal volume of the pseudovirus and incubated at 37 °C for 1 h. After incubation, the TZM-bl cell culture plates were removed from the incubator, the old culture medium was aspirated, and 100 µL of complete DMEM containing 80 µg/mL DEAE-Dextran was added to each well. The virus-serum mixture was then added to the plates (100 µL/well), with two replicate wells per dilution. Virus control wells (virus only) and cell control wells (cells only) were included. The plates were incubated at 37 °C with 5% CO_2_ for 48 h. Following incubation, the cells were lysed, and luciferase activity (Relative Luminescence Units, RLU) was quantified using the Bright-Glo Luciferase Assay System (following the manufacturer’s protocol). The mean RLU values for the sample wells (S), cell control wells (C), and virus control wells (V) were recorded. The neutralization percentage was calculated as [1 − (S − C)/(V − C)] × 100%. The serum neutralizing antibody titer was defined as the reciprocal of the highest serum dilution achieving 50% neutralization. The neutralization assay was performed using a single CRF01_AE pseudovirus. This approach allows for the sensitive detection of neutralizing activity against this homologous strain but does not assess the breadth of neutralization across diverse HIV-1 variants.

### 2.9. Enzyme Linked Immunospot (ELISpot) Assay

The frequency of Env-specific IFN-γ-secreting cells was assessed using the IFN-γ ELISpot assay kit (Mabtech AB, Nacka, Sweden) according to the manufacturer’s instructions. Briefly, mouse splenic lymphocytes were isolated using a mouse lymphocyte separation medium (Dakewei, China). Freshly isolated splenic lymphocytes were seeded at a density of 2 × 10^5^ cells/well in 96-well plates pre-coated with purified anti-mouse IFN-γ monoclonal antibodies. The cells were stimulated with H-2d-restricted Env-specific cytotoxic T lymphocyte (CTL) epitope peptides (for splenic lymphocytes), with each condition tested in triplicate (The peptides used in the ELISpot assay were selected from a peptide pool spanning the full-length gp145 immunogen. A total of 189 overlapping peptides were synthesized and dissolved in DMSO to prepare stock solutions at a concentration of 5 mg/mL each. Through preliminary experiments, we identified 11 peptides that were sensitive in BALB/c mice. This set of peptides was pooled for use in the ELISpot assay, with a final working concentration of 2 μg/mL per peptide). The cells were cultured for 48 h at 37 °C under 5% CO_2_. Negative controls consisted of cells cultured in 1% dimethyl sulfoxide (DMSO; Sigma-Aldrich, St. Louis, MO, USA), while positive controls were stimulated with 25 ng/mL phorbol myristate acetate (PMA) and 1 μg/mL ionomycin (Sigma-Aldrich). After incubation, spots were developed following the kit protocol. The plates were air-dried, and spot-forming cells (SFCs) were quantified using an ImmunoSpot^®^ Reader (CTL, Cleveland, OH, USA). A peptide-specific IFN-γ ELISpot response was considered positive if the response was at least four-fold higher than the negative control, and the SFCs exceeded 50 per 10^6^ splenic lymphocytes.

### 2.10. Data Analysis

The magnitude of humoral immune responses was expressed as the geometric mean titer (GMT) of HIV Gp120 binding antibodies or neutralizing antibodies in mouse sera. Antigen-specific cellular immune responses, measured by ELISpot assay, were reported as the mean number of HIV Env-specific IFN-γ spot-forming cells (SFCs) per 10^6^ splenic lymphocytes or. All data are presented as mean +/− the standard error of the mean (SEM). Statistical analyses were performed using GraphPad Prism (version 7.0; GraphPad Software, La Jolla, CA, USA). Data were assessed for normality using the Shapiro–Wilk test and for homogeneity of variances using the Brown–Forsythe/Levene-type test. All data met these assumptions; therefore, two-way ANOVA was used. Following significant main effects or interactions, Šídák’s post hoc test was performed to control the family-wise error rate. All tests were two-sided, with *p* < 0.05 considered statistically significant.

## 3. Results

### 3.1. Identification and Electron Microscopic Characterization of Recombinant Virus Particles Expressing HIV-1 AE gp145

**Figure 3:**
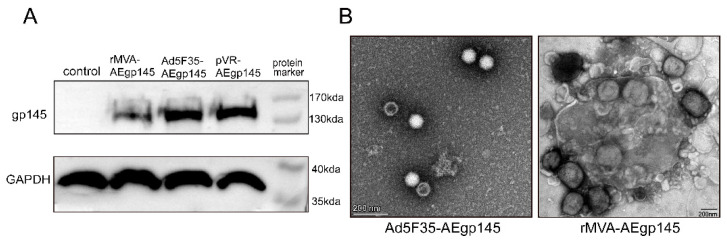
Characterization of HIV vaccines: (**A**) Analysis of HIV Env protein expression in HEK293 cells infected with Ad5F35-AEgp145 and BHK-21 cells infected with rMVA-AEgp145 (Positive control: pVR-AEgp145 (~145 kDa). Detection mode: chemiluminescence; exposure times: 10 s for the gp145 protein and 5 s for GAPDH). (**B**) Electron microscopy images of Recombinant virus particle.

As illustrated in Figure 1, the adenovirus vector vaccine and the vaccinia vector vaccine, both expressing HIV-1 GP145, were successfully constructed. Figure 3A demonstrates that HEK293 cells infected with recombinant viruses Ad5F35-AEgp145, and BHK-21 cells infected with recombinant viruses rMVA-AEgp145, exhibited distinct protein bands (130–180 kDa) corresponding to the expected size of HIV-1 GP145.

Electron microscopy analysis (Figure 3B) revealed virus particles with characteristic morphologies: approximately 300 nm for poxviruses (rMVA-AEgp145) and 70 nm for adenoviruses (Ad5F35-AEgp145), consistent with their respective viral families.

### 3.2. Specific Antibody Levels Induced by Different Vaccine Regimens: Homologous Ad5F35 Versus Heterologous rMVA Prime/Ad5F35 Boost

**Figure 4:**
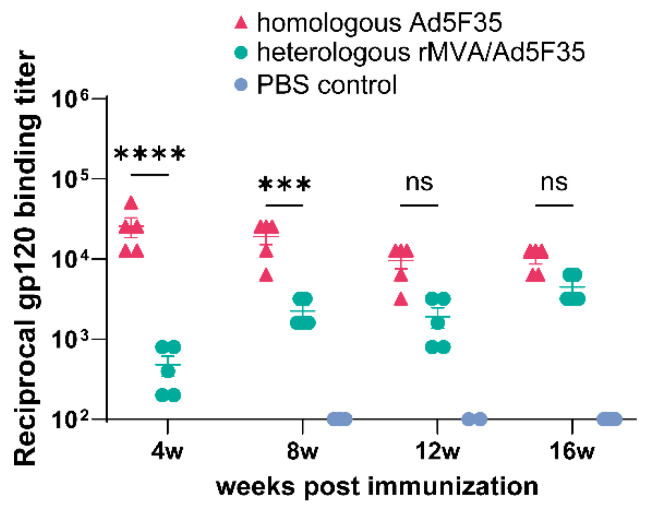
HIV-1 gp120-specific IgG titers in sera were detected by indirect ELISA. Error bars indicate ±SEM. *** *p* < 0.001; **** *p* < 0.0001; ns, no significant difference.

**Figure 5:**
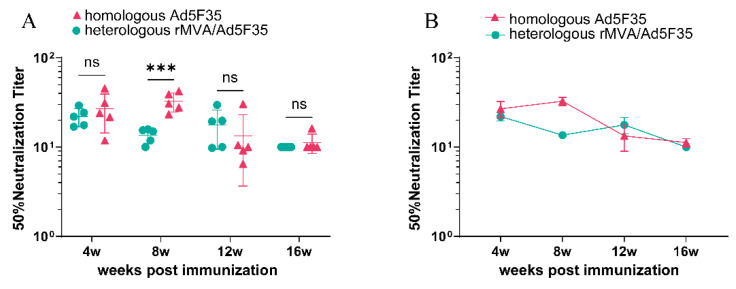
(**A**,**B**) 50% neutralizing antibody titers (NT50) against HIV-1 pseudovirus strain AE14 in sera. Error bars indicate ±SEM. *** *p* < 0.001; ns, no significant difference.

Mice in homologous Ad5F35 group were primed with Ad5F35-AEgp145 (10^6^ PFU) at week 0 and boosted with the same vaccine (equal dose) at week 3. Mice in heterologous rMVA/Ad5F35 group received rMVA-AEgp145 (10^6^ PFU) prime at week 0 followed by Ad5F35-AEgp145 (10^6^ PFU) boost at week 3. Mice in control group were administered two 100 μL PBS injections at corresponding time points. As shown in Figure 4, the highest levels of HIV-1 AE-gp120 specific antibodies were induced in the homologous Ad5F35 group, peaking at 4 weeks post the initial immunization with a GMT of 1:25,600. In contrast, the heterologous rMVA/Ad5F35 group showed a delayed peak response at 16 weeks post-primary immunization, reaching a GMT of 1:4480. Throughout the observation period, antibody titers in both groups remained within the range of 10^3^ to 10^5^. And the statistical significance for all key comparisons was shown in Figure 4 and Figure 5.

### 3.3. The Titers of Neutralizing Antibodies Elicited by Homologous Ad5F35 and Heterologous rMVA/Ad5F35 Vector Vaccines

Using a pseudovirus-based neutralization assay, we detected subtype AE-specific neutralizing antibodies in both vaccine groups at 4 weeks post the initial immunization. As shown in Figure 5, at 4 weeks after the initial immunization, the homologous Ad5F35 group showed a 50% neutralization titer (NT50) of 1:24, compared to 1:22 in the heterologous rMVA/Ad5F35 group. At week 8, the homologous Ad5F35 group showed higher neutralizing antibody levels, which were higher than those in the heterologous group. The 50% neutralization titer was 1:45 in the homologous group and 1:12 in the heterologous group (*p* < 0.01). The neutralizing antibody titers elicited by homologous and heterologous vector vaccines exhibited a subtle downward trend over time. At the 16th week post-vaccination, neutralizing antibodies remained detectable in these two groups, with titers of 1:12 and 1:10, respectively, while no specific neutralizing antibodies were detected in the serum of PBS control group at any time point.

### 3.4. Robust Env-Specific Cellular Immune Responses Were Detected in Vaccinated Mice by IFN-γ ELISpot Assay

**Figure 6:**
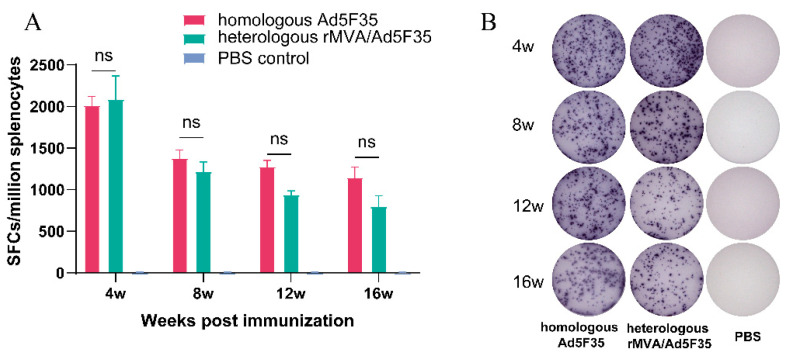
Cellular immune responses in splenocytes assessed by IFN-γ ELISpot. (**B**) Freshly isolated splenic lymphocytes (5 × 10^4^/well) were stimulated in duplicate wells with Env-specific peptides (2 μg/mL), and (**A**) the IFN-γ secreting cells were quantified as mean spot-forming cells (SFCs) per 10^6^ lymphocytes. Error bars indicate ±SEM. ns, no significant difference.

In the mice immunized with homologous Ad5F35 and heterologous rMVA/Ad5F35 vector HIV vaccines, IFN-γ producing lymphocytes peaked at week 4 after initial immunization, reaching 2020 and 2090 SFCs per 10^6^ lymphocytes, respectively. This demonstrates that both strategies effectively elicited robust cellular immune responses. Subsequently, response levels declined fluctuantly in both groups. By week 16, responses maintained relatively high, with 1245 SFCs/10^6^ lymphocytes in the homologous Ad5F35 group and 714 SFCs/10^6^ lymphocytes in the heterologous group, respectively (Figure 6).

## 4. Discussion

Our previous studies in mice and rhesus macaques demonstrated that sequential immunization with multi-vector vaccines expressing HIV-1 gag can induce high levels of antigen-specific cellular immunity. Substantial evidence confirms that vaccine-elicited HIV-specific cellular immune responses correlate with viral replication control [26]. In this study, HIV-1 AEgp145 vaccines based on Ad5F35 and rMVA viral vectors were successfully constructed, and their immunogenicity was systematically evaluated in a mouse model. The results indicated that Ad5F35 vector alone group and rMVA prime-Ad5F35 boost group could effectively trigger specific humoral and cellular immune responses against HIV-1 Env. This finding is consistent with the results of our previous research on the Ad5F35/rMVA vaccine expressing SIV surface proteins in mice [27].

Regarding humoral immunity, at weeks 4 and 8 after the initial immunization, the Ad5F35 alone group induced significantly higher levels of gp120-specific binding antibodies compared to the heterologous rMVA/Ad5F35 prime-boost group. This phenomenon might be associated with the inherent properties of the vectors and the immunization intervals. As an adenoviral vector, Ad5F35 is well-known for its efficient gene delivery capabilities and robust immune-activating potential [28]. Employing the homologous immunization strategy for consecutive immunizations might have led to a more robust antibody response by repeatedly stimulating and expanding memory B cells specific to the antigen expressed by the same vector. In contrast to the weak humoral immune response induced by the rMVA vector vaccine alone in our previous studies, the homologous Ad5F35 immunization regimen induced higher levels of HIV-1-specific binding antibodies. This finding provides strong support for the development of multi-vector combined immunization strategies.

Based on previous research conducted by our team (data unpublished), the truncated Env antigen gp145 (with the gp41 cytoplasmic tail removed) were selected rather than the full-length gp160 in this study. The mice experiment results indicate that the pVR-AE gp145 vaccine induced significantly stronger cellular and humoral immune responses than the pVR-AE gp160. The cellular immune response peaked at 3 weeks and 4 weeks post immunization, respectively, whereas binding antibody peaked at 3 weeks post-last immunization in both group. These results indicated that the truncated gp145 was a superior target antigen by both maintaining T-cell epitope recognition and enhancing the immune response level. Consequently, it was selected for our HIV vaccine research.

Notably, despite the significant differences in the levels of binding antibodies induced by the two strategies, the levels of neutralizing antibodies they elicited were relatively comparable. This suggests that under the immunization protocol employed in this study, these two vector-based vaccines predominantly triggered immune responses against non-neutralizing antibody epitopes of the HIV Env protein. To induce high-quality antibodies that can effectively recognize and neutralize the virus by targeting crucial conformational epitopes, further optimization of immunogen design or the implementation of combinatorial immunization strategies involving other antigen forms may be indispensable.

Regarding cellular immunity, both strategies elicited robust Env-specific IFN-γ responses. These responses reached their peak 4 weeks after the primary immunization and were maintained at a relatively high level throughout the entire observation period. This finding is in line with the results of our research group’s previous work, in which an Ad5F35/rMVA vector-based SIV vaccine similarly induced a robust cellular immune response. Robust cellular immunity, particularly the CD8+ T cell response, has been well-established to be closely associated with the control of HIV/SIV viral replication [29]. The findings of this study further validate that the vaccine platforms based on Ad5F35, regardless of whether it is used alone or combined with rMVA, are effective approaches for eliciting anti-HIV cellular immunity.

As a vaccine based on the adenoviral vector (Ad5F35), the potent anti-adenoviral immune response it induces is a critical factor that must be considered when evaluating its application prospects. Although the Ad5F35 vector used in this study has been modified, potentially reducing cross-reactivity with pre-existing immunity against the common serotype 5 adenovirus (Ad5), the prime-boost strategy itself may still induce strong anti-Ad5F35 vector immunity [30,31]. This could limit the efficacy of the same vector in subsequent booster immunizations or affect its suitability as a platform for multiple booster regimens or universal vaccines. This study did not quantify anti-adenovirus antibody levels or vector-specific T-cell responses, thus preventing a direct assessment of their specific impact on the observed immune responses, particularly those following booster immunization.

Certainly, this study has several limitations. The experiments in this study were confined to a mouse model, which may not fully capture the complexity of human biology. The lack of validation in non-human primate models limits the generalizability of our findings to human populations. Future studies should incorporate non-human primates or other higher-order species to confirm the relevance and translatability of these results to humans. Although neutralizing antibodies were detectable, the analysis was limited to a single homologous pseudovirus and did not assess breadth or potency against diverse viral variants. Further investigations are therefore required to assess the neutralization capacity of these antibodies against diverse viral strains, including newly emerging variants, to better define their potential as a broadly protective measure. Collectively, these limitations indicate that the present preclinical findings remain preliminary and underscore the need for follow-up studies in more advanced animal models, ultimately progressing to phased clinical trials to rigorously evaluate vaccine safety and efficacy.

## 5. Conclusions

This study demonstrates that the Ad5F35-AEgp145 vaccine, whether administered alone or in combination with rMVA-AEgp145, is capable of eliciting specific humoral and cellular immune responses. The homologous Ad5F35 regimen demonstrated an advantage in eliciting higher binding antibody titers, while both strategies elicited comparable T-cell responses. Furthermore, the neutralization potency observed in this study was modest, and no breadth assessment against diverse viral strains was performed. Therefore, these results should be regarded as preliminary, highlighting that further optimization of immunization strategies remains essential for achieving synergistic, high-quality antibody and T-cell responses critical for an effective HIV vaccine. Future work will prioritize evaluating the protective efficacy of these strategies in non-human primate challenge models and characterizing the durability and quality of the induced immune memory.

## Data Availability

All data related to this study are included in this article.
